# Tailored PCL Scaffolds as Skin Substitutes Using Sacrificial PVP Fibers and Collagen/Chitosan Blends

**DOI:** 10.3390/ijms21072311

**Published:** 2020-03-27

**Authors:** Ali Reza Sadeghi-avalshahr, Samira Nokhasteh, Amir Mahdi Molavi, Najmeh Mohammad-pour, Mohammad Sadeghi

**Affiliations:** 1 Department of Materials Research, Iranian Academic Center for Education, Culture and Research (ACECR), Mashhad Branch, Mashhad 91775-1376, Iran; Sadeghi_av@ymail.com (A.R.S.-a.); samira.nokhaste@gmail.com (S.N.); 2 Department of Biomaterials, College of Biomedical Engineering, Amirkabir University of Technology, Tehran 159163-4311, Iran; 3 Materials Engineering Department, Tarbiat Modares University, Tehran 14115-111, Iran; 4 Department of Stem Cell and Regenerative Medicine Research, Iranian Academic Center for Education, Culture and Research (ACECR), Mashhad Branch, Mashhad 91775-1376, Iran; najmeh.mohammadpoor@yahoo.com.sg; 5 Department of Mechanical Engineering, Tarbiat Modares University, Tehran 14115-111, Iran; H.sadeghi@ymail.com

**Keywords:** collagen, chitosan, aminolysis, electrospinning, grafting, wound healing

## Abstract

Electrospinning is a versatile technique for fabrication of made-on-purpose biomimetic scaffolds. In this study, optimized electrospun fibrous membranes were produced by simultaneous electrospinning of polycaprolactone (PCL) and polyvinylpyrrolidone (PVP), followed by the selective removal of PVP from the PCL/PVP mesh. After aminolysis, a blend of collagen/chitosan was grafted on the surface. Physicochemical characterizations as well as in vitro evaluations were conducted using different methods. Successful cell infiltration into samples was observed. It seems that the positive trend of cell ingress originates from the proper pore size obtained after removal of pvp (from 4.46 μm before immersion in water to 33.55 μm after immersion in water for 24 h). Furthermore, grafting the surface with the collagen/chitosan blend rendered the scaffolds more biocompatible with improved attachment and spreading of keratinocyte cell lines (HaCaT). Viability evaluation through MTT assay for HDF cells did not reveal any cytotoxic effects. Antibacterial assay with *Staphylococcus aureus* as Gram-positive and *Escherichia coli* as Gram-negative species corroborated the bactericidal effects of chitosan utilized in the composition of the coated blend. The results of in vitro studies along with physicochemical characterizations reflect the great potentials of the produced samples as scaffolds for application in skin tissue engineering.

## 1. Introduction

In recent decades, the growing trend of various skin wounds caused by chronic diseases, such as diabetic foot ulcers, pressure ulcers, etc., as well as acute wounds induced by burns or surgery has stimulated researchers to look for a suitable skin substitute. Some of the most important characteristics for an ideal skin substitute include safety (nontoxicity, non-immunogenicity, and non-carcinogenicity), proper adhesion, moisture permeability, water loss prevention, infection control, and desirable mechanical properties [1,2]. The imitation of the natural extracellular matrix (ECM) appears to offer the best method for fabrication of a scaffold as the skin substitute. There are two main features to ECM: the fibrous structure and the presence of natural proteins such as collagen, which consists of ~25–35% of the whole-body protein contents in the extracellular matrices of many fibrous tissues of mammals including tendons, skin, bones, and so on. So far, 29 types of collagen have been discovered and classified. Among them, type I collagen forms over 90% of the collagen of the body.

This fibrous structure of ECM can be mimicked via electrospinning and subsequent coating by natural proteins [3,4,5].

Among different methods for fabricating skin substitutes including freeze-drying, phase inversion, 3D printing, and laser-assisted techniques, electrospinning has received special attention, mainly due to its ability to produce nano- and microfibrous structures with high a surface-to-volume ratio [4,6], which could be effective in cell–scaffold interactions.

A rather broad range of synthetic and natural polymers have been utilized for the fabrication of skin substitutes using electrospinning technique. They include collagen, gelatin, chitosan, silk fibroin, poly(L–lactide) (PLLA), poly(lactide–*co*– glycolide) (PLGA), polycaprolactone (PCL), as well as various blends of these materials [4]. PCL was used in this study for its biodegradability, biocompatibility, and relatively good mechanical properties, such as high plasticity, ease of formation by various techniques, shrinkage-free structure in aqueous solutions, and its low cost [4,7,8,9]. The major drawback of PCL, however, is its hydrophobic nature and the absence of cellular recognition sites in its surface microstructure [7,8,9], which may lead to poor cell–surface interactions [10]. Many attempts have been made to improve the surface hydrophilicity of PCL by different techniques. The main methods used for surface modification of PCL include plasma treatment, chemical treatment, coating by natural proteins, and blending in bioactive components [7]. Each of these methods has its own advantages and drawbacks. Physical adsorption and covalent bonding are two possible routes for the coating of scaffolds [11]. Safaeijavan et al. provided some gelatin-coated scaffolds of electrospun PCL after oxygen plasma treatment, and concluded that the proliferation rate and viability of fibroblast cells improved after the surface coating of scaffolds [10]. Sharif et al. grafted collagen onto PCL scaffolds after plasma treatment, observing that the attachment and proliferation rate of human endometrial stem cells improved in comparison with the pristine PCL scaffolds [12]. Ghosal et al. prepared collagen-coated scaffolds of PCL by immersing scaffolds in collagen solutions to enhance their hydrophilicity [13]. In another study, Croisier et al. produced some electrospun scaffolds of PCL and poly(MMA-MA). After coating scaffolds with the chitosan through electrostatic interactions, they did not observe any growth of bacterial colonies in the chitosan-coated mats [14]. Ezhilarasu et al. investigated loaded PCL/aloe Vera (AV) blends with curcumin (CUR) and tetracycline hydrochloride (TCH), separately, using electrospinning technique to compare their effects and reported better antibacterial activity and surface wettability for PCL/TCH in comparison with PCL/AL, which could be helpful in wound healing applications [15].

One of the frequently used methods for surface functionalization of polyester-based scaffolds is the aminolysis reaction, which introduces free amine groups on the surface of polymers [16]. The synthesis of COOH-functionalized PCL nanofibers through alkaline hydrolysis is another well-known method for its surface modifications [17,18].

In this work, after aminolysis on the surface of the electrospun PCL scaffolds, samples were coated with a blend of two biopolymers—chitosan and collagen—using the covalent bonding through different functional groups of PCL such as carbonyl (C=O) to form amide bonds (N-C=O). It is hypothesized that the two features of antibacterial nature and desirable cell signaling could be achieved by the newly introduced cell recognition sites in this approach. A number of physical, mechanical, and biological characterizations were carried out to evaluate the final produced scaffolds.

## 2. Results

### 2.1. Microstructural Observations

**Figure 1:**
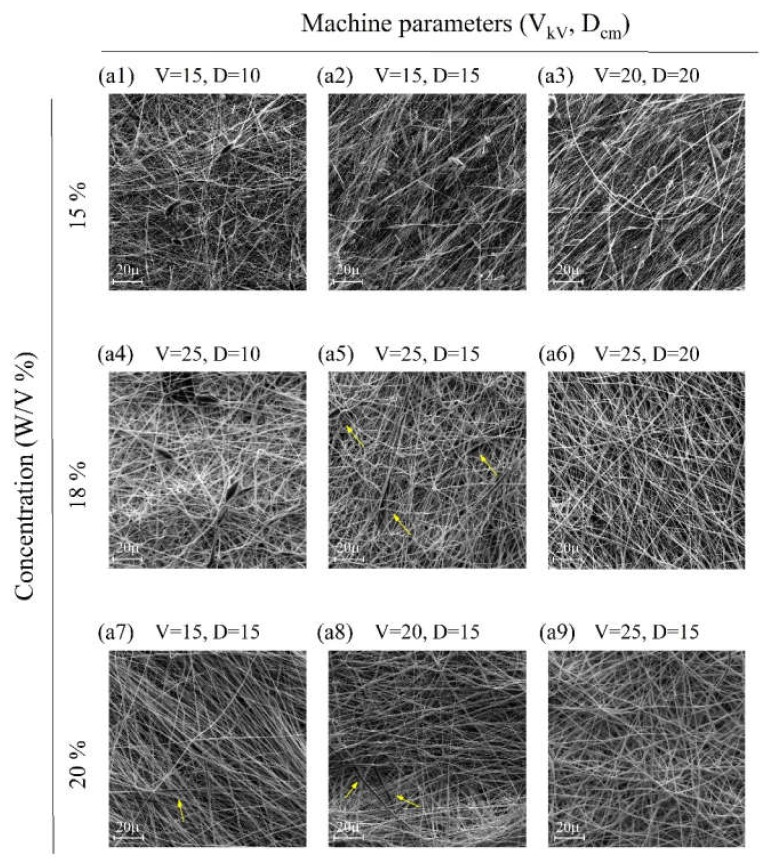
(**a1**–**a9**) SEM images showing the morphology of as-spun polycaprolactone (PCL) fibrous mats obtained from solutions at different concentrations and various machine parameters (V and D) (arrows show some beads have formed in microstructures).

Figure 1 shows the results of PCL electrospinning at various concentrations in the acetic acid (90% *v*/*v*). Figure 1a9 was observed to offer the best condition for obtaining a beadless structure (PCL concentration of 20% *w*/*v*, V = 25 KV and D= 15 cm).

**Figure 2:**
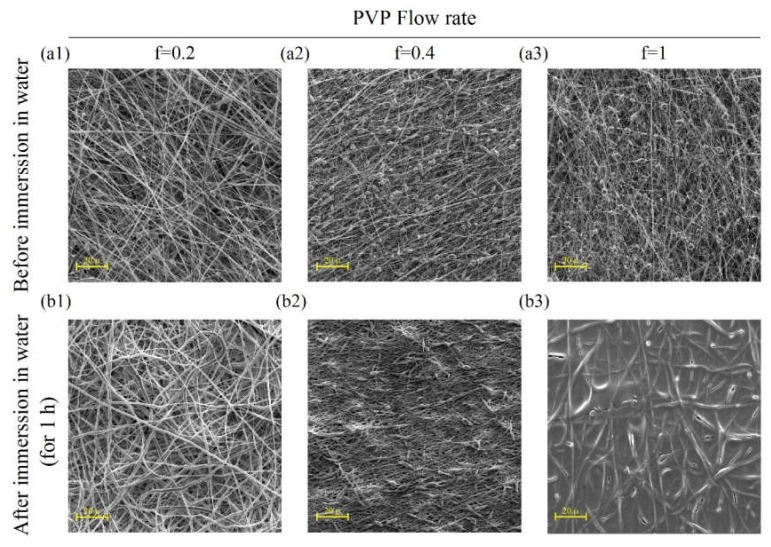
SEM images showing micrographs of co-electrospun PCL (20% *w*/v, V = 25 KV and D= 15 cm) with polyvinylpyrrolidone (PVP) (25% *w*/*v*, V = 25 KV and D= 15 cm) by various flow rates for PVP (ml/h): (**a1**–**a3**) before and (**b1**–**b3**) after immersion in water for 1h.

As can be seen, given the tight structure of the fibers in Figure 1, we attempted to use some sacrificial fibers to increase the pore size of the microstructure for improved cellular infiltration [19,20]. In this work, PVP was chosen and utilized as the sacrificial fiber due to its rather desirable solubility in water for simultaneous spinning (25% *w*/*v*, V = 25 KV and D= 15 cm). The prepared solutions of PVP and PCL, in compliance with the above-mentioned conditions, were electrospun using two opposite and independent spinnerets of different flow rates (0.2, 0.4, and 1 mL/h for PVP and 1 ml/h for PCL). Electrospinning was continued for 10 h to obtain samples with a final thickness of about 250 μm. Figure 2 shows the resultant microstructure of electrospun samples before and after immersion in water for 1 h. After soaking specimens in water, PVP fibers were dissolved gradually and the pore size increased.

**Figure 3:**
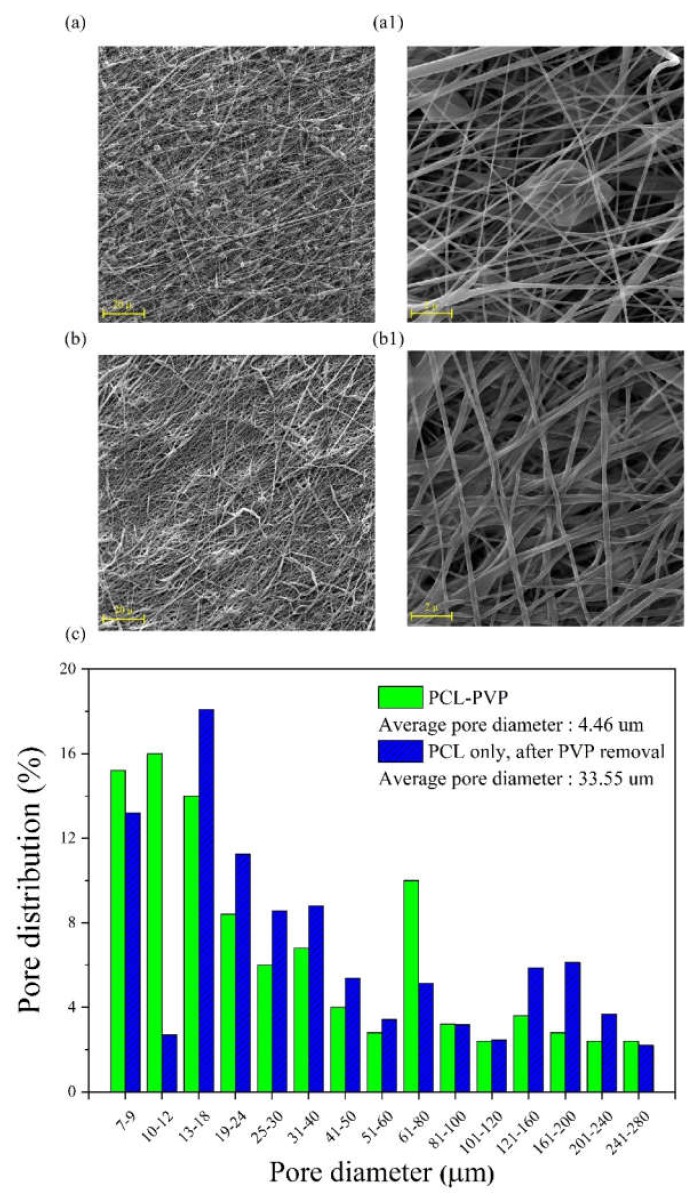
SEM images of PCL fibrous mats after the soaking of simultaneous electrospun PCL/PVP samples in water for 24 h: (**a,a1**) before immersion; (**b,b1**) after immersion; (**c**) comparative histograms for pore diameter distribution before (PCL-PVP)and after (PCL) immersion in water.

However, a balance should be maintained between the preservation of structural integrity and the decreasing of microstructural density. Therefore, a flow rate of 0.4 mL/h was selected for the next stage. It seems that beads, as shown in Figure 2a1–a3, are related to PVP fibers, as they disappeared partially after immersion in water as it displayed in Figure 2b1–b3. However, there has not been sufficient time for them to be completely dissolved. As depicted in Figure 3, the increasing of the immersion time to 24 h at the next step led to complete dissolving of PVP fibers (Figure 3b,3b1) with significant increase in the average pore diameter of samples (from 4.46 μm before immersion to 33.55 μm after immersion in water, Figure 3c).

**Figure 4:**
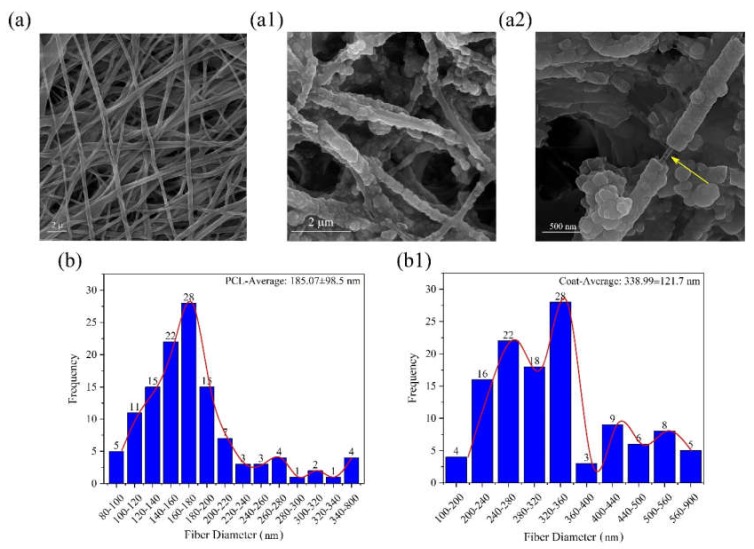
(**a**) SEM morphology of pristine PCL (control); (**a1**,**a2**) SEM morphology of aminolized PCL electrospun mats grafted with collagen/chitosan blend under different magnifications (arrow shows a typical uncoated part); (**b**,**b1**) comparative histograms of fiber diameter before (b) and after (b1) coating.

A recognized approach for grafting biopolymers onto the polyester surface of PCL is to introduce some functional groups such as primary amine groups (NH_2_) onto the surface using the aminolysis technique [21,22]. These amine groups could be utilized for grafting collagen/chitosan blend onto the aminolized surface of PCL through different functional groups of PCL such as carbonyl (C=O) to form amide bonds (N-C=O) [23]. The microstructure of samples after coating with a blend of collagen/chitosan is shown in Figure 4.

**Figure 5:**
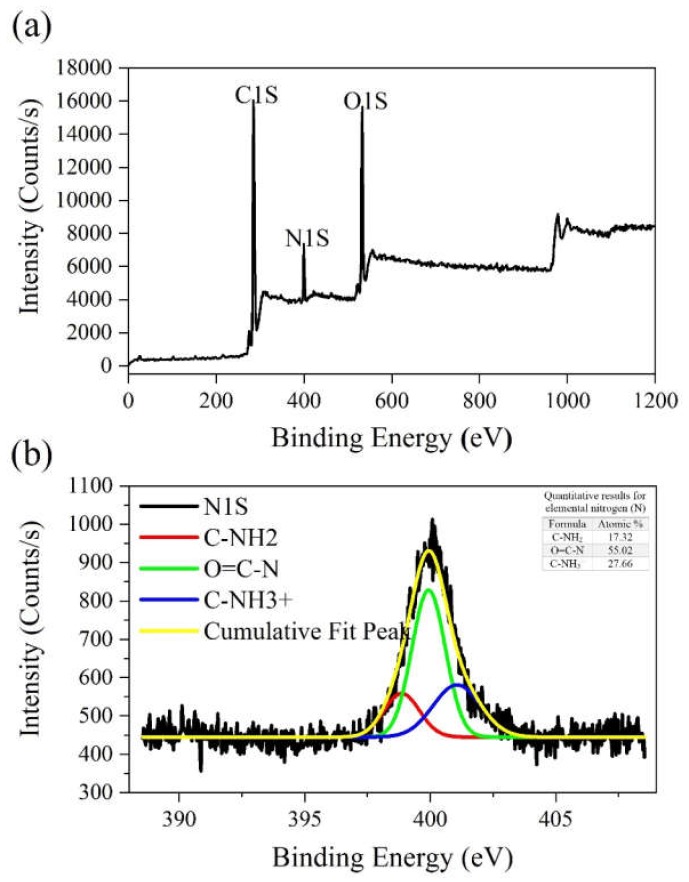
The results of X-ray photoelectron spectroscopy (XPS) spectra analysis: (**a**) Collagen/chitosan-grafted PCL membranes; (**b**) detailed and quantitative analysis of elemental nitrogen (N1s).

Both smooth and granular coating areas could be observed in the microstructure (Figure 4a–a2). A comparison of this image with the previous one (Figure 3) reveals that the coating process was conducted successfully. As displayed in Figure 4b,b1, the average diameter of fibers is ~185 nm and 339 nm before and after coating, respectively. Complementary evidence for the evaluation of coated surfaces is displayed in Figure 5, based on the results of XPS analysis. The N1s peak in Figure 5a and the detailed and quantitative analysis of this peak is shown in Figure 5b.

**Figure 6:**
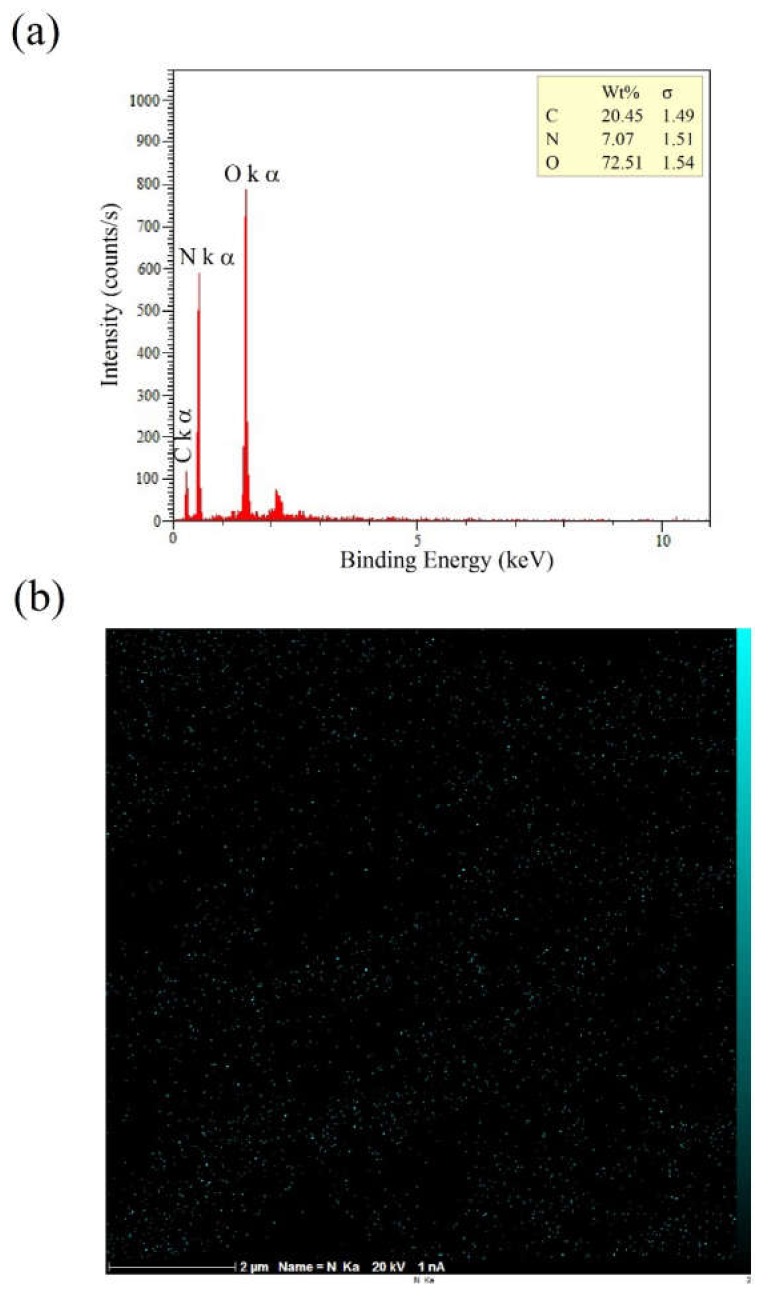
Energy-dispersive X-ray spectroscopy (EDXS) analysis results: (**a**) nitrogen-related peaks; (**b**) 2D elemental mapping of nitrogen.

Figure 6b depicts a uniform distribution of nitrogen gained from 2D elemental mapping of nitrogen (N) using EDXS analysis and related peaks (Figure 6a), which reaffirms the success of the coating process. These findings suggest that the coating process has been carried out successfully.

### 2.2. Mechanical Properties

**Figure 7:**
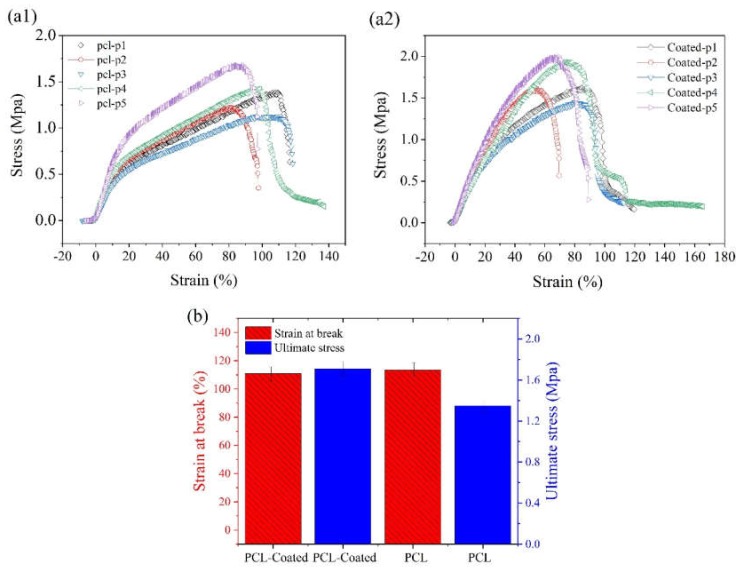
**(a1,a2)** Stress–strain curves of (**a1**) pristine PCL membranes and (**a2**) collagen/chitosan-coated specimens; (**b**) Comparative histograms of ultimate stress and strain for pristine PCL (PCL-p1 to p5) and coated specimens (coated: p1–p5) (The comparison of data between the two groups (*n* = 5) using one-way ANOVA did not show any significant difference for *p* < 0.05.).

The stress–strain curve of the PCL electrospun samples before and after coating with collagen/chitosan blend are shown in Figure 7a1,a2 respectively. The related histograms of ultimate stress as well as strain at break are displayed in Figure 7b. The results of data comparison with one-way ANOVA did not reflect any significant difference between these two groups in terms of ultimate tensile stress (UTS) and strain at break (*p* < 0.05, *n* = 5).

### 2.3. In Vitro Evaluations

#### 2.3.1. Cell Attachment

**Figure 8:**
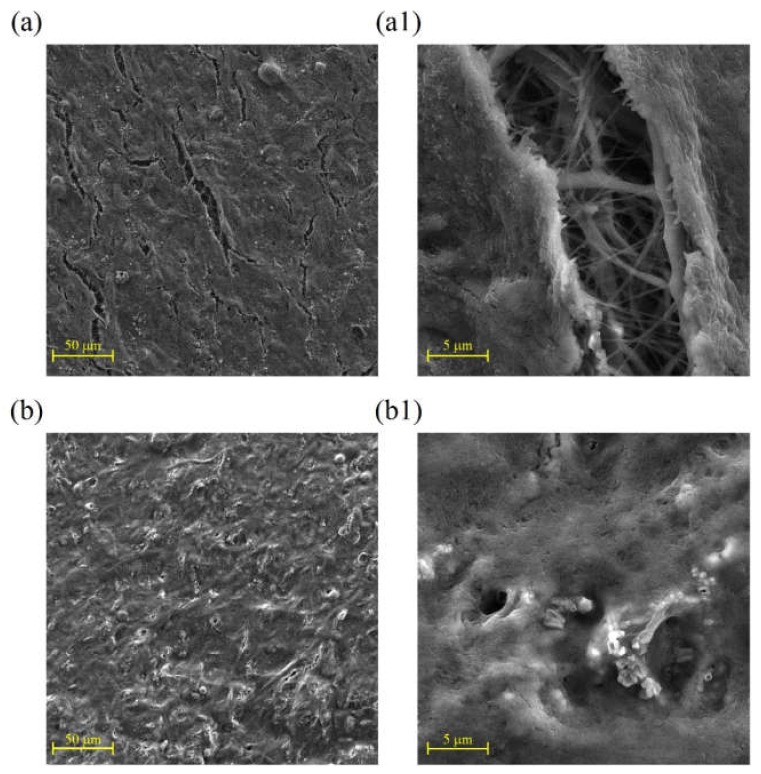
SEM images showing 3T3 fibroblast cell line adhesion on (**a**,**a1**) the pristine PCL electrospun samples; (**b**,**b1**) collagen/chitosan grafted specimens (cultured for 3 days).

**Figure 9:**
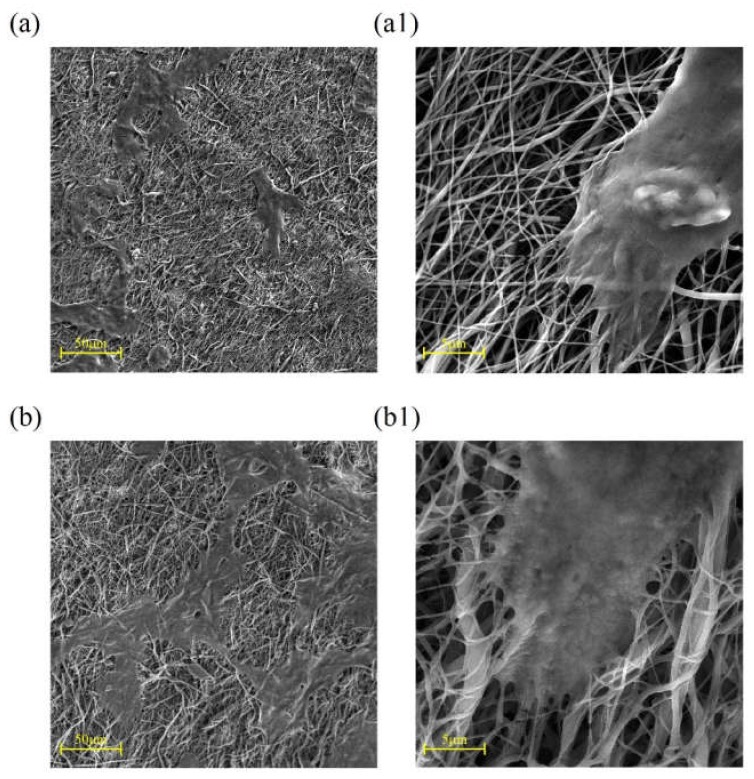
SEM images showing HaCaT cell line adhesion on (**a**,**a1**) pristine PCL electrospun samples; (**b**,**b1**) collagen/chitosan grafted specimens (cultured for 3 days).

SEM images of cell attachment for 3T3 fibroblast and keratinocyte (HaCaT) cell lines after culturing for three days are shown in Figure 8 and Figure 9, respectively.

Due to the strong binding ability of the fibroblast cells to both substrates in Figure 8, the effect of surface modification of samples through collagen/chitosan coating on cell attachment could not be evaluated properly using this cell line. Thus, the fibroblast cell line was replaced with the HaCaT cell line for next assay. As it can be seen in Figure 9, using the HaCaT cell line can represent improvement of cell–surface interaction due to coating of samples.

#### 2.3.2. MTT Assay

**Figure 10:**
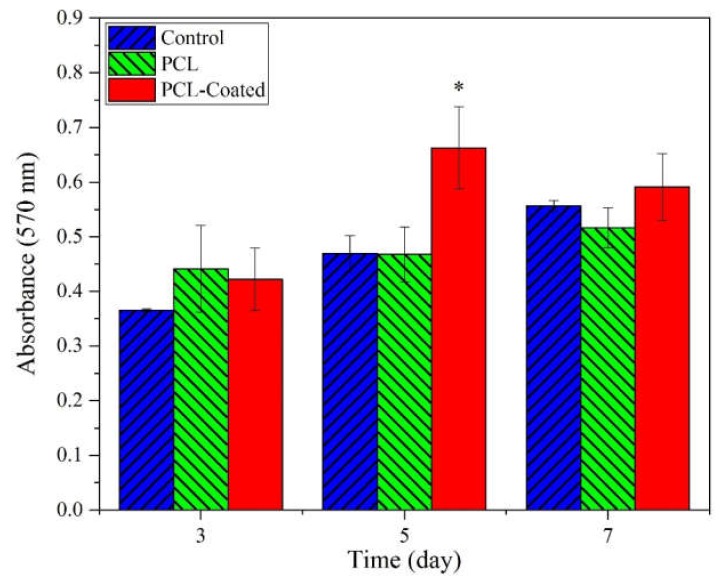
Evaluating the viability of HDF cells on samples using MTT assay at different times (3, 5, 7 days) for pristine PCL fibrous mats and collagen/chitosan coated samples. Medium without scaffold was considered as the control. (One-way ANOVA analysis indicated a statistically significant difference between coated and uncoated specimens at day 5, * *p* < 0.05, *n* = 3.)

The proliferation rate of the HDF fibroblasts in the as-spun and coated PCL samples was estimated by MTT assay after 3, 5, and 7 days, as described in Section 4.4.5. The data is plotted in Figure 10. The level of significance was obtained from one-way ANOVA. A significantly high cell proliferation rate was observed in coated samples in comparison with the control and as-spun samples after 5 days (*p* < 0.05, *n* = 3).

#### 2.3.3. Cell Infiltration

**Figure 11:**
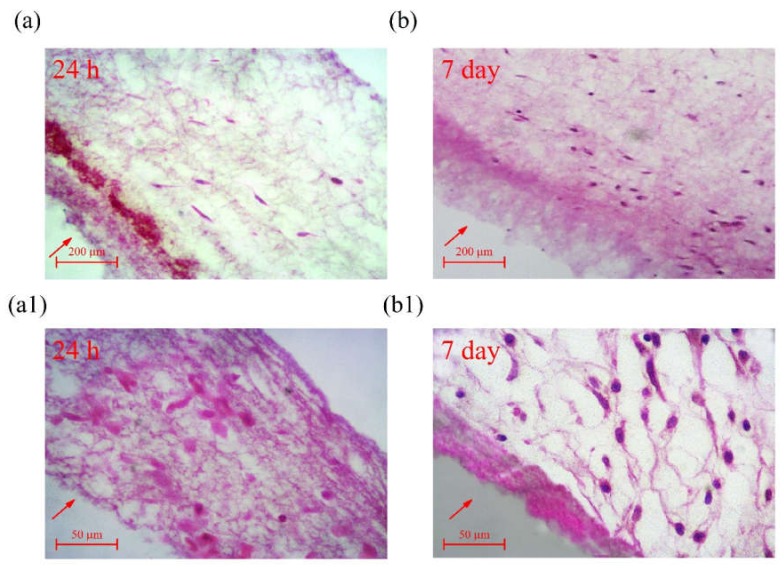
Evaluation of cell ingress into PCL fibrous mats using HDF cells: (**a**,**a1**) after 24 h; (**b**,**b1**) after 7 days (after simultaneous electrospinning of PVP and PCL, PVP fibers were removed to increase the porosity of electrospun samples).

A comparison of cross sections of samples in Figure 11a,b after 24 h and 7 days reveals a positive trend for the infiltration of HDF cells into electrospun PCL scaffolds from the top to underneath layers of the surface, representing the proper pore size (Section 2.1) and architecture of fabricated samples for cellular ingress.

#### 2.3.4. Antibacterial Assay

**Figure 12:**
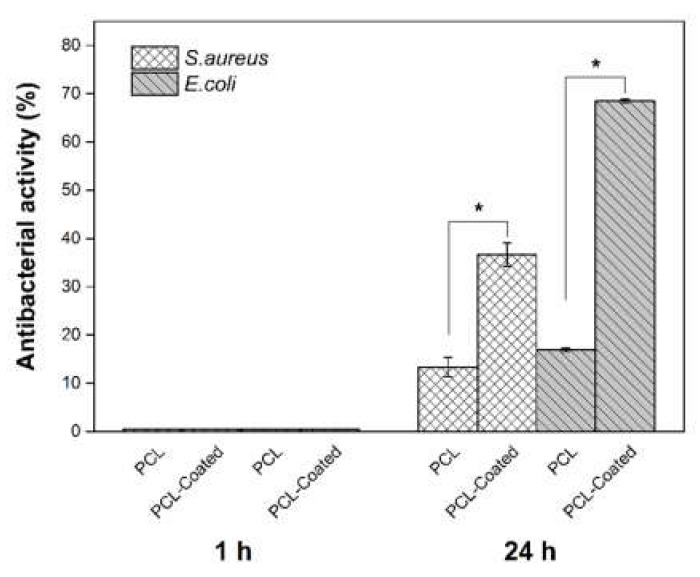
Histograms of antibacterial assay against two bacterium strains for pristine PCL and coated scaffolds after 1 h and 24 h. (One-way ANOVA analysis indicated a statistically significant difference between the antibacterial activity of coated samples in comparison with uncoated ones after 24 h, * *p* < 0.05, *n* = 3.)

As shown in Table 1 and Figure 12, in both bacterial strains, collagen/chitosan-coated scaffolds revealed significant improvement in their antibacterial activities after 24 h, in comparison with pristine PCL specimens. In addition, according to the results, the antibacterial effects of coated samples against *S. aureus* are lower than that of *E. coli* after 24 h contact time. Also, no antibacterial effect was observed at the first experiment (1h) in both samples.

## 3. Discussion

There are two major factors involved in achieving good cell–scaffold interactions: the scaffold architecture and its chemical composition. These two factors exert synergetic effects on providing a successful bioactive scaffold for tissue engineering applications. Electrospinning is a versatile technique to produce a fibrous structure for the mimicking of ECM. However, as far as the architecture is concerned, a balance should be maintained between cell adhesion, which is more properly conducted by the sub-micron size of structural fibers, and cell infiltration, which prefers a pore size arising from a micron-order structure [24,25]. In this study, PCL, as a common polymer that is used in biomedical engineering applications, was utilized; however, due to its hydrophobic nature and the absence of cell recognition sites, it is not a good choice for tissue engineering applications and requires surface modification for improved biocompatibility. Different authors have studied the incorporation of major biopolymers in PCL through coating or blending. It is conducted through collagen [12,13,23,26], or its denatured form, gelatin [10,27], and chitosan as a natural polysaccharide [14,28,29], or a blend of chitosan–gelatin [30]. In this work, a blend of both collagen and chitosan was coated onto the surface of electrospun PCL samples, and, to the best of the author’s knowledge, this is the first study to use them as a blend for grafting on electrospun PCL mats. It was hypothesized that this approach capitalizes on two features of improved cell–surface interaction and antibacterial activity. Due to toxic effects of GA, the least possible amount of GA was used for the aminolysis process, because after coating of the collagen/chitosan blend, it was expected that the intermolecular interactions could occur by hydrogen bonds formation through –OH and –NH_2_ groups as well as –C= O groups in collagen with –OH groups and –NH_2_ groups in chitosan. Also, ionic bonds maybe formed between anionic –COOH groups in collagen and chitosan, which is a cationic polysaccharide and finally form polyanionic–polycationic complex [31,32].

To achieve an optimized and fairly beadless microstructure for primary electrospun PCL scaffolds, three steps were taken sequentially in different experiments, as mentioned before. First, the best PCL concentration was achieved for proper spinnability (i.e., having proper solubility, viscosity, surface tension, and jet stability). The most effective parameters of the electrospinning machine such as voltage and distance were chosen. Finally, PVP was used as a sacrificial polymer to electrospun simultaneously with PCL to gain a proper final pore size and fiber density in the microstructure (Figure 1 and Figure 2). A proper ratio of PVP/PCL was selected by establishing compromise between preserving integrity of microstructure after the removal of PVP fibers and achieving a proper pore size in the final microstructure (Figure 3).

Figure 5a shows three peaks at approximately 285eV, 400eV, and 532 eV relative to C1s, N1s, and O1s, respectively. The elemental nitrogen (N), which was detected by XPS analysis, indicates the successful introduction of amine groups onto the coated surface of samples (due to absence of nitrogen in the chemical structure of PCL). Detailed and quantitative analysis of the cumulative curve of N1s that has put as an inset in Figure 5b confirms the formation of amide bonds (O=C-N, ~55% atomic) between carbonyl groups of PCL and amine groups (NH_2_) introduced to its surface. Also, the C-NH_2_ bond (~17.3% atomic) may reflect the presence of chitosan onto the modified surface of PCL (due to absence of this bond in the chemical structure of PCL and collagen). Considering the fiber microstructure in Figure 4 (as compared to Figure 3) also reaffirms the deposition of collagen/chitosan blend onto the electrospun surfaces. Moreover, regarding the ultimate tensile strength and elongation at break in Figure 7a1, a2 and b, it seems that smooth coating of PCL fibers by collagen/chitosan blend has failed to induce a significant change in their mechanical properties, probably due to the intact backbone of the microstructure (which are PCL fibers in both states, before and after coating).

Fibroblasts and keratinocytes are two major cells, which play a pivotal role in wound healing process. In this study, they were utilized to investigate cell attachment and cytocompatibility. Figure 9 depicts improved cell adhesion for HaCaT on coated scaffolds in comparison with the pristine PCL samples. It seems that coating with collagen has facilitated the adsorption of serum proteins onto the modified surfaces probably due to enhancement of hydrophilicity. This factor, along with the cationic nature of chitosan in the coated blend composition, has contributed to the spread and adhesion of cells on the coated surfaces. In fact, these adsorbed serum proteins, by supporting the focal adhesion complexes, activate various cell–matrix recognition pathways, and thereby improve cell adhesion and spread onto the coated samples [33]. Figure 10 shows elevated absorption (at 570 nm) for coated scaffolds after 5 and 7 day of cell culturing, which represents superior cell proliferation. This improvement is significantly higher at day 5, in comparison with the control and pristine PCL. This may be due to the small unwanted change in pH in favor of acidification after 7 days or may be due to conquer the rate of cell proliferation relative to rate of cell migration and infiltration into underneath layers of scaffold that can yields in cellular over-confluency on the surface of scaffolds which in turn could has negative effect on cells viability at the end of day 7. It appears that the presence of amino acid sequences of collagen on the coated surfaces provides proper cell recognition sites for improved cell adhesion and proliferation, which in turn expedites the wound healing [29]. The relative range of typical rounded cell size is 5–20 µm [34]. Some authors have argued that the pore size of a scaffold should be at least proportionate to the cell size [35]. However, others have maintained that stem cells can ingress inside scaffolds even through small pore sizes by pushing aside the nanofibers web [36]. Densely packed fibers in the microstructure of electrospun samples and the subcellular space between fibers (structural pore size) could lead to the hindrance of cell infiltration into the scaffold [37]. Several strategies have been proposed to address these shortcomings in the electrospinning technique including increased fiber diameter, combination of micro- and nano-scale fibers, and utilization of sacrificial fibers or particles, to mention a few [20,38,39].

Utilization of PVP as sacrificial fibers and its removal after electrospinning has reduced fiber density and provided a proper pore size for cellular infiltration (Section 2.1), which, as it can be seen in Figure 11, has resulted in successful infiltration of HDF cells into the underneath layers of electrospun samples.

Selected bacteria for antibacterial assays in this work (*S. aureus* and *E. coli*) are two of the most important species, which play key roles in the inducing of infection at wound sites. Gram-positive bacteria such as *S. aureus* appear in the initial phase of wound construction, and Gram-negative species including *E. coli* are present in the advanced phases of wound healing, especially in chronic wounds [40,41,42]. According to the results of Table 1 and Figure 12, coated scaffolds (sample B) against *E. coli* appear to have superior antibacterial effects in comparison with *S. aureus* after 24h. This is in consistent with the findings of our previous study [36] as well as results reported in other studies [43,44,45]. It seems that the high hydrophilicity of Gram-negative bacteria in comparison with Gram-positive species renders them more susceptible to the membrane damage via chitosan cations. In this process, changing their membrane permeability results in leakage and drainage of cellular proteins, which disrupts their functions at the end.

## 4. Material and Methods

### 4.1. Materials

Chitosan (high molecular weight; degree of deacetylation = 85%) and phosphate-buffered saline (PBS) were obtained from Sigma-Aldrich. Bovine collagen type I (AteloCollagen) with molecular weight of 300 KDa was purchased from Wuxi BIOT Biology Technology Co. LTD (china). Glacial acetic acid and 2-propanol (99.5%) were purchased from Dr. Mojallali Co. (Iran). PCL (M_n_= 80000), Polyvinylpyrrolidone (PVP) (M_w_= 360000), glutaradialdehyde (GA) 25%, and 1,6- Hexanediamine (HDA) were provided by Merck. Ethanol (99.8%) was purchased from Simin Taak. Co (Iran).

### 4.2. Electrospinning

#### 4.2.1. Solution Preparation

According to the process proposed by Li et al. [46], PCL with various concentrations of 15%, 18%, and 20% (*w*/*v*) was prepared by dissolving in the glacial acetic acid. Solutions were then mixed with gentle magnetic stirring for 24 h at room temperature. The required deionized water was added to each homogeneous solution after 24 h of stirring to reach 90% *v*/*v* acetic acid and then stirred for an additional 30 min. Also, PVP with 25% *w*/*v* was dissolved in ethanol and stirred gently for 2 h.

#### 4.2.2. Fiber Formation via Electrospinning

Electrospinning of PCL solutions was conducted by varying the most effective parameters of the machine (HV35P OV, Fanavaran Nano-Meghyas, Iran) including nozzle-to-collector distance (D) and voltage (V). The collector speed was set at 800rpm and considered to be constant throughout the experiments. Electrospinning was performed at room temperature with relative humidity of ~20–25% using the needle gauge 22. The best results, which required achieving a beadless structure, were chosen for the simultaneous electrospinning of PVP and PCL using two opposite and independent spinnerets of varying flow rates. Electrospun samples were soaked in water afterward for various times to dissolve the PVP fibers and the best result was selected for preparing the final samples.

### 4.3. Surface Modification of Electrospun Samples

#### 4.3.1. Preparation of the Biopolymers Solution

According to our previous experiments as well as the work of Zhu et al. [22], chitosan with concentration of 3 mg/mL was dissolved in 0.5M acetic acid and magnet stirred gently at 60 °C for 3 h. Moreover, 5 mg/mL collagen was dissolved in 0.5M acetic acid and stirred gently at room temperature for 24 h. The collagen and acetic acid were mixed in a 1:1 volume ratio and stirred for an additional 2 h to prepare the final coating solution.

#### 4.3.2. Aminolysis and Coating

Surface aminolysis of the electrospun PCL samples was carried out in accordance with the process proposed by Zhu et al. [22]. In brief, this process is as follows; electrospun samples are immersed in an alcohol/water solution (1/1, *v*/*v*) for 2 h to be cleaned and then washed by copious deionized water. Then, for functionalization of the surface with amine groups, the samples are immersed in HDA/2-propanol solution (1:1, *v*/*v*) with a concentration of 10%wt at 37 °C in a refrigerated shaking incubator (SHER300, Noorsanat, Iran) for 45 min. In the next step, the samples are washed in deionized water for 24 h and dried at 30 °C. The aminolized samples are immersed in the GA solution (1% *w*/*v*), as coupling agent, at room temperature for 3 h to improve the stabilization of biopolymers. Then, the samples are washed thoroughly with deionized water 4 times to remove any free GA. Finally, the treated samples are immersed in a collagen/chitosan solution for 24 h at 2–4 °C followed by immersion in the deionized water for 30 min, twice washing, and then drying.

### 4.4. Characterization

#### 4.4.1. Morphological Observations

The morphological microstructure of samples was studied using field-emission scanning electron microscopy (FE-SEM) (Mira3tescan-XMU, Czech Republic). Image processing was conducted using ImageJ software (1.47 v) for the measurement of fiber diameters. At least 100 fibers were used for measurement, and the average fiber diameter as well as distribution of fiber size was reported.

#### 4.4.2. Porosimetry

The porous structure of samples was studied using a PASCAL-140 mercury porosimeter.

#### 4.4.3. X-ray Photoelectron Spectroscopy (XPS)

X-ray photoelectron spectroscopic (XPS) spectra were recorded on a BESTEC (Germany) spectrometer using Al Kα excitation radiation (X-ray source powered at 20 mA and 15 kV) and a vacuum system of 10^−10^ mbar. The maximum lateral dimension of the analyzed area was 10 mm.

#### 4.4.4. Tensile Test

The mechanical behavior of the electrospun and coated samples (stress–strain curves, ultimate stress, and strain at break) was studied using a microtensile test machine (model TA Plus, USA) at room temperature. Five rectangular pieces (3 × 1 cm) from each pristine and coated PCL samples were provided for the tests. A load cell of 50 N and a crosshead speed of 10 mm/min were chosen for the tests.

#### 4.4.5. Cell Attachment

Circular discs of coated samples (22 mm diameter) and pristine samples of electrospun PCL (as control) were prepared and sterilized by UV for 60 min on each side. To ensure that the cell broth does not leak under the samples attached to the surface of culture plates, samples were fixed with some type of fixtures similar to cell crown inserts (custom made). They were then disinfected with ethanol (70% v) and finally sterilized by UV for an additional 20 min on each side. In the next step, they were seeded in triplicates of 3 × 10^4^ cm^−2^ with keratinocyte (HaCaT) and 3T3 fibroblast cell lines (immortalized primary cell lines), separately, and cultured with each for three days. Samples were then washed twice with PBS to remove the unattached cells. Attached cells were fixed using 2.5% glutaraldehyde for 3 h. After being rinsed in double distilled water and dried under a laminar hood, samples were evaluated using a field-emission scanning electron microscope (Mira3tescan-XMU, Czech Republic). Note that due to the more sensitive nature of HaCaT cells compared with 3T3 fibroblasts, for better observation of the effect of surface modification on improvement of the cell–surface interactions, the HaCaT cell line was selected for the cell attachment test.

#### 4.4.6. MTT Assay

The cytotoxicity of the scaffolds was evaluated through the MTT assay using a direct contact method. To do so, samples were sterilized and fixed in cell crowns as stated in the previous section and placed in 12-well plates in triplicate. Human dermal fibroblast primary cell lines (HDF) (3 × 10^4^ cm^−2^), which play key roles in wound healing, were chosen for the assay and were seeded onto each sample and cultured in DMEM with 100 U mL^−1^ penicillin/100 µg mL^−1^ streptomycin and 10% FBS. The medium was refreshed every three days. Moreover, 50 μL of MTT solution with a concentration of 5 mg/mL was added to each well and plates were incubated subsequently at 37 °C for 3 h. After incubation, the MTT reaction medium was substituted with 600 μL/well dimethyl sulfoxide (DMSO)/isopropanol. MTT assay was carried out on days 3, 5, and 7 and the medium without scaffold was utilized as the control. The absorbance was measured at 570 nm using Elisa plate Reader (model ELX808, BioTeK, USA). Absorbance value of each sample was calculated after the subtraction of optical density (OD) from the blank sample (scaffold without cells).

#### 4.4.7. Cell Infiltration

The cell ingress into the fibrous scaffolds was evaluated using the histological process for cellularized scaffolds. To do so, after seeding samples with 3 × 10^5^/well of HDF cells in 24-well plates in triplicate for 24, 48, and 72 h, they were fixed in 4% paraformaldehyde for 2h at room temperature. Following dehydration with graded alcohols and clearing, they were embedded in paraffin and sectioned at 6μm thickness using a microtome. Staining was performed by hematoxylin and eosin (H&E) and stained sections were observed under light microscopy (LABOMED, Los Angeles, USA) and photographed by a DeltaPix camera.

#### 4.4.8. Antibacterial Assay

Two bacterial strains were used at the logarithmic stage of growth: *S. aureus* (ATCC 6538, PTCC 1112) was used as Gram-positive and *E. coli* (ATCC 25922, PTCC 1399) as Gram-negative species. The antibacterial activity was evaluated in triplicate in accordance to the method proposed by Ko et al. [47]. Samples were sterilized by UV for 30 min prior to the experiments. In this method, 500 μL of bacterial suspension (1.5 × 10^6^ CFU/mL) was placed on each prepared circular sample (22 mm in diameter) and then samples were incubated for 1 and 24 h at 37 °C. The samples were then placed into some sterile plastic tubes, diluted with PBS, and homogenized by being centrifuged at 10,000 rpm for 1 min. The final solution was diluted by PBS decimally (from 10^−1^ to 10^−5^), cultured on the plate count agar and incubated at 37 °C for 48 h. The enumeration of viable bacteria was undertaken and compared with the control (pristine uncoated samples of PCL). Antibacterial activity was calculated according to the following formula.

(1) $$\mathrm{Antibacterial}\;\mathrm{activity}\;(\%)=\frac{control\left(CFU/ml\right)-sample\left(CFU/ml\right)}{control\left(CFU/ml\right)}\times 100$$

## 5. Conclusions

In this study, fibrous scaffolds were produced through co-electrospinning of PCL with PVP as the sacrificial polymer. Machine parameters and solution concentration were chosen in such a way to achieve an acceptable and rather beadless microstructure. The results of porosimetry along with the cellular infiltration assay confirmed the effectiveness of adopting PVP as sacrificial fibers for increasing microstructural pore size. The surface modification of electrospun samples through aminolysis and grafting by collagen/chitosan blend improved the surface biocompatibility, as demonstrated by the in vitro assay. Evaluation of the antibacterial activity of modified samples elucidated the role of chitosan in coated blend regarding in terms of its bactericidal effects. A striking result was that using a blend of chitosan and collagen for grafting onto the surface yielded good cell–surface interaction and desirable bactericidal effects. This offers promising potentials for wound healing process as the skin regenerating scaffolds.

## Figures and Tables

**Table 1:** Results of antibacterial assay against two bacterium strains for A (pristine PCL) and B (coated scaffolds) in three iterations (the blank control for all samples was 1.5 × 10^6^ CFU/mL).

Microorganism	Contact time (h)	Sample	(CFU/mL)	Antibacterial Activity (%)
*E. coli* (ATCC25922)	1	A	1.5 × 10^6^	no antibacterial activity
		B	1.5 × 10^6^	no antibacterial activity
	24	A	1.25 × 10^6^	16.66
		B	4.7 × 10^5^	68.66
*S. aureus* (ATCC6538)	1	A	1.5 × 10^6^	no antibacterial activity
		B	1.5 × 10^6^	no antibacterial activity
	24	A	1.3 × 10^6^	13.33
		B	9.5 × 10^5^	36.66
